# Three-Dimensional Rotational Angiography in Pediatric Patients with Congenital Heart Disease: A Literature Review

**DOI:** 10.1007/s00246-019-02052-z

**Published:** 2019-01-24

**Authors:** Femke van der Stelt, Sebastiaan N. Siegerink, Gregor J. Krings, Mirella M. C. Molenschot, Johannes M. P. J. Breur

**Affiliations:** Department of Pediatric Cardiology, Wilhelmina Children’s Hospital, University Medical Center Utrecht, Lundlaan 6, P.O. Box 85090, 3508 AB Utrecht, the Netherlands

**Keywords:** Cardiac catheterization, Congenital heart disease, Pediatric, Review, Rotational angiography

## Abstract

Cardiac catheterization is a commonly used form of imaging and treatment in pediatric patients with congenital heart disease. Traditionally, two-dimensional conventional angiography was the method used, but since 2000 three-dimensional rotational angiography (3DRA) is increasingly used in the field of cardiology in both adult and pediatric patients. To investigate the use and applications of 3DRA in pediatric congenital cardiology, literature was systematically reviewed and 29 eligible articles were found. Those showed that 3DRA is already a greatly valued diagnostic and therapeutic technique in pediatric cardiology. However, the literature misses well-designed clinical, homogeneous, multicenter, prospective studies recording data in a standardized manner. These studies are necessary to ensure proper data analysis and to investigate the true advantages of 3DRA and how it exactly benefits the patients.

## Introduction

Traditionally, two-dimensional conventional angiography (CA) is the method used to visualize and percutaneously treat congenital heart diseases (CHD). While three-dimensional rotational angiography (3DRA) was already a well-established technique in neurology [1], it was only in 2001 that Boccalandro and colleagues reported the first application of 3DRA in an adult patient with congenital heart disease [2]. In this patient, computed tomography showed a thoracic aneurysm after coarctectomy with side-to-side graft placement for aortic coarctation. Magnetic resonance angiography and CA could not solve the patients’ anatomic enigma as opposed to 3DRA. The rotational aortogram with reconstructed 3D model revealed close proximity of the distal aortic stump and graft giving the impression of dilatation in the repaired portion of the aorta. The patient was discharged without intervention [2].

Today, 3DRA is more widely used in the field of cardiology among both adult and pediatric patients [3–5]. This paper will function as a literature review of the current research literature on 3DRA in pediatric patients with congenital heart disease and will summarize the current applications and results of this technique in this patient group. Furthermore recommendations to improve research in this field are given.

## Methods

Relevant articles were selected from the PubMed library and EMBASE, with the latest search on 28-11-2017. The following keywords were used in combination: 3DRA, three-dimensional rotational angiography, cardiology, cardiac, and heart. Studies were included if they matched up with the following criteria: 3DRA was used to evaluate or treat congenital heart defects and the studied patients had a mean or median age lower than 18 years. Studies were excluded in case of phantom data, animal data, ablation or cardiac resynchronization therapy, coronary angiography, 3DRA used for non-cardiac purposes, imaging other than 3DRA, congress abstracts, general reviews on 3DRA, and editorial comments. Data of interest for our review were as follows: type of congenital heart defect, application of technique (e.g., diagnostic evaluation, stent intervention, percutaneous pulmonary valve implantation (PPVI)), vendor used, radiation dose, contrast dose, side effects, and complications.

## Results

### Study Selection

**Fig. 1:**
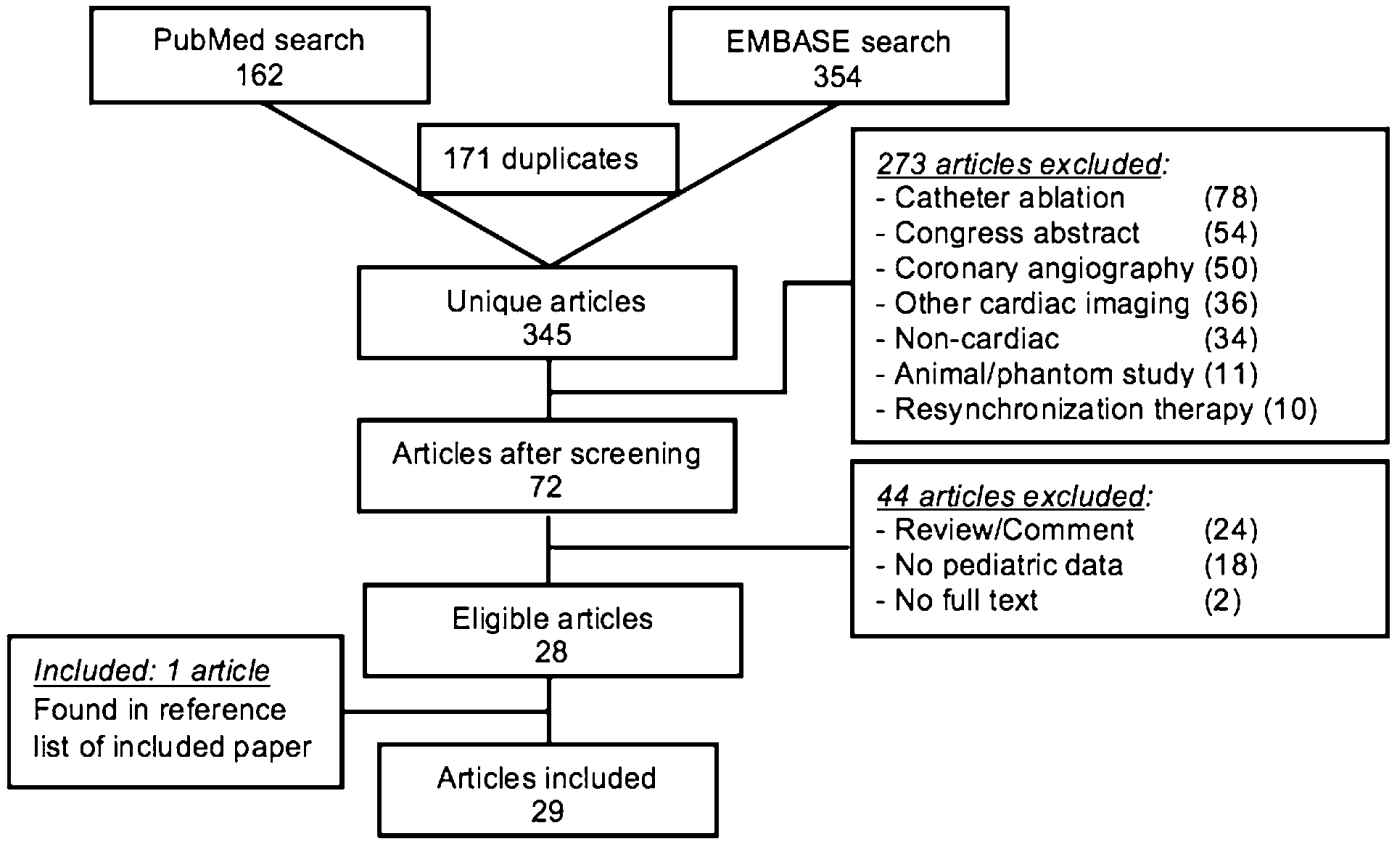
Flowchart of literature search and study selection

A total of 516 articles were found, of which 345 were unique (Fig. 1). Based on screening of title and abstract, 273 articles were excluded. Of the remaining 72 articles, 28 were eligible for our review. One additional study was found by searching reference lists. Seven of the included articles were case reports, and ten studies described data of both children and adults with congenital heart disease.

### Reason of Catheterization and Sample Sizes

The articles are listed in Table 1 with details on catheterization indication, number of patients/studies, age, radiation dosages, and vendor used. The reason of catheterization was diverse; five studies were for sole diagnostic reasons (e.g., evaluation of cavopulmonary connection), 12 studies were interventional (e.g., PPVI or coarctation therapy), and 12 were combined diagnostic and interventional. Within 11 studies, reason of catheterization was even miscellaneous. Sample sizes for 3DRA varied from one till 109 patients. Three different imaging vendors were used: Siemens, Philips, and Toshiba.

**Table 1:** Details on catheterization indication, number of patients/studies, age, and radiation of the included articles

Author, year [references]	Diagnostic/interventional; indication	Number of studies/patients	Age (median, range)	DAP µGy/m^2^	Other radiation value	Vendor
Aldoss 2016 [6]	Diagnostic; miscellaneous	114/87	2.7 years (1 day–48.4 years)	One 3DRA run 72.3 (4.4–779.0)	No	Philips
Anderson 2015 [7]	Interventional; aorta stenosis	1	15 years	No	AK 3DRA runs 151 and 143 mGy; total 977 mGy	Siemens
Berman 2012 [8]	Both; CPC	37/32	4.3 years (0.3–19)	One 3DRA run: 306 (100–5902); CA 159 (42–2102); total 1525 (364–43,557)	No	Toshiba
Borik 2015 [9]	Diagnostic; post-CPC airway/vasculature	25	Mean 3.1 ± 2.0 years	One 3DRA run 245 (65–1038) CA 178 (49–3566)	3DRA run AK 11 (6–38) mGy, CA 21 (5–390) mGy	Siemens
Corredoira 2015 [10]	Both; miscellaneous	170/109	7.5 years (0–19)	One 3DRA run 238 (35.0–4240.3)	No	Siemens
Ebrahim 2015 [11]	Interventional; bronchoscopy guidance LPA stenting	4/3	9–49 months	No	No	Toshiba
Glatz 2010 [12]	Both; miscellaneous	41	5.1 years (0.4–58.8)	No (phantom data)	No	Siemens
Glöckler 2011 [13]	Both; miscellaneous	62	3.5 years (0–42.5)	One 3DRA run 111.0 (19.3–1295.7); total 341.9 (37.6–7249.7)	No	Siemens
Glöckler 2013 [14]	Interventional; miscellaneous	61	9.6 years (0–42.5)	One 3DRA run 164.0 (38.6–1276.6); total 706.3 (104.8–7249.7)	No	Siemens
Glöckler 2013 [14]^a^	Interventional; stenting aortic coarctation^a^	12 3D, 20 CA	3D 15.6 years (13.5–19.5), CA 14.5 years (12.6–16.8)	Total DAP 3D 1429.6 (832.9–2067.2); CA 1942.0 (1415.5–2929.5)	No	Siemens
Glöckler 2013 [15]	Both; CPC	31	1.9 years (0.3–42.5)	One 3DRA run 91.8 (33–679.3); total 228.7 (33.3–7249.7)	No	Siemens
Goreczny 2016 [16]	International; PDA closure	1	12 months	No	No	Philips
Goreczny 2016 [17]	Interventional; ductal stenting HLHS	11 3D runs in 6 patients; 12 CA	20 days (13–31)	One 3DRA run 16 (12.4–22.5); total DAP 3D 263.7 (147.4–519.5), CA 507.7 (259.0–1491.6)	No	Philips
Goreczny 2017 [18]	Interventional; PPVI	6 3D; 8 CA	CA 14 years (9.7–19.6); 3DRA 13.8 (12.3–17.6)	Total 3DRA 10,823.3 (5961.2–15,265.9); CA 17,745.9 (13,411.2–24,808.5)	AK 3DRA 727 (400.1–1024.6) mGy, CA 1191 (900.1–1665) mGy	Philips
Haddad 2016 [19]	Both; miscellaneous	100 3D; 100 CA	3DRA 10.2 years (1.12–43.87); CA 9.96 years (0.33–39.52)	One 3DRA run 278 (107–595); CA 241 (124–760); total 3DRA 3605 (1679–18,033); CA 3544 (1186–10,761)	ED one 3DRA run 1.8 (1.2–2.8) mSv, CA 1.67 (1.08–3.7) mSv; AK: total 3D 250 (146–816) mGy and CA 265 (121–531) mGy	Toshiba
Hill 2013 [20]	Interventional; closure Fontan fenestration	1	5 years	No	No	Siemens
Kapins 2010 [21]	Both; miscellaneous	53	6 years	One 3DRA run 374.5 ± 228.1; CA 356.5 ± 327.4	AK 3D 41,467 ± 27,561 mGy, CA 30,019 ± 27,516 mGy	Philips
Manica 2014 [22]	Diagnostic; miscellaneous	18	12.5 years (1–44)	Total 3DRA 1093 (701–1767); CA 360 (200–1049)	AK population 171 (40.6–1767) mGy	Siemens
Moszura 2013 [23]	Interventional; middle aortic syndrome	1	3.5 years	No	No	Siemens
Nguyen 2016 [24]	Interventional; PPVI/Melody	29 3D; 52 CA	3DRA 17.92 years (10–48); CA 24.67 years (5–57)	Total 3DRA 7765.81 (1373.01–42,945.46); CA 6546.66 (822.28–60,928)	No	Toshiba
Panzer 2008 [25]	Diagnostic; coronary	1	2.5 years	No	AK 101 mGy	Unknown
Patel 2013 [26]	Diagnostic; double aortic arch	1	18 days	No	No	Toshiba
Peters 2015 [27]	Both; miscellaneous	17/14	5.7 years (0–16)	No	ED 1.6 (0.7–4.9) mSv	Siemens
Pockett 2017 [28]	Both; PPVI candidacy	31	3–58 years	No	No	Toshiba
Poterucha 2014 [29]	Interventional; PPVI	1	15 years	No	No	Siemens
Starmans 2016 [3]	Interventional; aortic coarctation	42 3D (15 balloon, 27 stent), 104 CA (61 balloon, 43 stent)	Balloon 3DRA 0.32 (0.25–2.91) years, CA 0.60 (0.28–1.26) years; stent 3DRA 12.82 (8.78–14.76) years, CA 9.1 (3.43–13.34) years	All DAPs are in µGy/m^2^/kg Balloon 3DRA run 5.97(3.01–8.16), 3D + CA run 8.61 (6.71–14.06); stent 3DRA run 22.17(15.23–30.54), 3DRA + CA run 22.31(8.11–34.71) Total balloon 3DRA 15.81 (6.97–44.70), 3D + CA 22.52 (16.17–45.09), CA 27.88 (16.12–44.11); stent 3DRA 45.24 (37.38–81.34), 3D + CA 48.90 (36.04–107.25), CA 37.34 (25.93–59.77)	No	Siemens
Stenger 2016 [4]	Interventional; aorta	50	0.02–39.2 years	Total 3DRA 139 (27–1305)	No	Siemens
Stenger 2016 [4]^a^	Interventional; aorta^a^	31 3D, 20 CA	3DRA 13.9 years (10.2–17.8), CA 14.5 years (12.6–16.8)	Total 3DRA 839 (532–1838); CA 1942 (1416–2930)	No	Siemens
Surendran 2017 [30]	Both; miscellaneous	15	15 months (4–24)	One 3DRA run 128 (68–141); total 442 (162–746)	ED 3D run 1.35 (0.67–1.78) mSv; AK one 3D run 11.4 (8.2–18.6) mGy, AK total 95 (38–157) mGy	Toshiba
Truong 2015 [31]	Both; airway	8	2.5 years (5 weeks–7 years)	Total 3DRA 85 (47.5–224.9)	ED 0.16 (0.09–0.42) mSv	Philips
Zahn 2011 [32]	Both; miscellaneous	7	7 days–10 years	No	No	Toshiba

*AK* air kerma, *CA* conventional angiography, *CPC* cavopulmonary connection, *DAP* dose area product, *ED* effective dose, *HLHS* hypoplastic left heart syndrome, *LPA* left pulmonary artery, *PDA* patent ductus arteriosus, *PPVI* percutaneous pulmonary valve implantation, *3D* three-dimensional, *3DRA* three-dimensional rotational angiography

^a^Subanalysis of data from the article

### Benefits of 3DRA

Most articles described the diagnostic qualities of 3DRA as being superior to that of conventional angiography. 3DRA visualized the complex anatomy in detail prior to surgical or catheter-based interventions [3, 6, 8, 12, 13, 20, 21, 24, 26, 28], including the anatomy of the surrounding tissues (e.g. airway) [9, 11, 15, 31] and it has the ability to view the anatomy from unlimited angulations [8, 15, 32]. In addition, interventions were performed in a faster and safer way [14], as the obtained 3D images were used as a roadmap for intervention guidance [4, 7, 13, 16, 17, 19, 20, 23, 24].

### Possible Negative Effects

Radiation data, dose area product (DAP), effective dose (ED), or air kerma (AK), were mentioned in 17 studies, of which 11 compared 3DRA with CA. Factors influencing radiation such as patient weight, amount of contrast used, and fluoroscopy time were mentioned in 27, 20, and 12 studies, respectively. Table 1 shows that some studies reported high radiation dosages with 3DRA when compared to CA [8, 22], whereas other studies found similar [3, 9, 14, 19, 21, 24] or lower radiation dosages [4, 17, 18].

Seven studies mentioned whether or not complications occurred during catheterization. In six of these, no complications or serious adverse events occurred [11, 16, 21, 23, 29]. Starmans *et al*. report the complications that occurred and describe a transient right bundle branch block after right ventricular pacing in one patient [3]. The other complications could not be related to 3DRA.

## Discussion

After introduction of the technique in the field of cardiology in 2001, 3DRA is increasingly used in adult and pediatric patients with CHD. This literature review collates the current applications and results of 3DRA in pediatric CHD. The main message of the 29 eligible articles is that 3DRA provides detailed information of both vasculature and surrounding tissues and it can be performed in a fast and safe way. Besides, it optimizes interventions as the images can be used as guidance for interventions and it overcomes limitations seen with CA (e.g., unlimited angulations). However, some studies report high radiation dosages when compared to CA and state that reduction measurement should be taken, whereas other studies find similar or lower radiation dosages. These results show that 3DRA is a promising imaging technique, which is still developing in the field of pediatric cardiology. However, there is room for improvement in the research performed and this will be discussed below.

The diagnostic quality of 3DRA was described as ‘superior,’ ‘extremely helpful,’ and as ‘providing information not usually seen by CA’[6, 21, 23]. Scoring of image quality was not solely objective and differed among the studies. One article gave a definition of image quality [3] and two studies correlated vessel diameters measured on 3DRA with corresponding CA images [4, 6, 8, 9, 13, 22]. Other studies used a modified Likert scale [4, 15] or similar score [6] to describe if the information obtained with 3DRA was ‘essential,’ ‘very useful,’ ‘useful,’ ‘not useful,’ or ‘misleading’ when compared to CA. In the other articles, it was not clear where ‘being of diagnostic quality’ was based on [8, 12, 13]. It is desirable to make a clear definition of image quality and how it should be assessed, apart from ranking the usefulness of the information obtained.

3DRA revealed more irregularities in the anatomy than CA and thus required additional interventions [8, 12, 13]. These defects would otherwise have gone unnoticed [18]. In addition, detailed visualization of cardiovascular anatomy and surrounding tissues is necessary to evaluate whether patients are suitable for intervention or not (e.g., PPVI or pulmonary artery stenting). This could reduce possible unexpected complications as coronary artery compression post-PPVI or bronchial compression after pulmonary artery stenting [8, 28]. Another advantage of 3DRA is the possibility to use the obtained 3D images as an overlay onto live fluoroscopy to guide percutaneous interventions [7, 12, 14, 17, 24]. This fastens and simplifies the interventions [4, 15].

Conversely, there were also some concerns about 3DRA. For example, higher radiation dosages, specifically in children, were expected [22]. A study designed to create a radiation protocol for the use of 3DRA in a pediatric cardiac catheterization laboratory intended to identify the radiation doses and contrast levels for children. The article proposed that 3DRA use is currently restricted due to the unknown risk of increased radiation exposure [19]. However, most articles reported that 3DRA also had equal [3, 9, 14, 19, 21, 24] or less contrast and radiation exposure when compared to CA, even when interventions were performed [4, 17, 18]. To add to that, some studies even indicate that the doses can be further diminished by reducing the frame rate and by getting better acquainted with the equipment [10, 22, 27]. Besides, many articles admitted to a learning curve causing higher contrast and radiation exposure at the start, which dropped after getting more familiar with the technique or after having consulted a technician from the corresponding 3DRA vendor [4, 6, 8, 9, 13, 22]. Starmans et al. showed that their 3DRA DAPs decreased over 50% over time [3]. This is an indication of both the vast differences between the first exposure and the optimized exposure to radiation, as well as the reduction that is possible when the system works optimally.

### Research Performed and Improvements

A few things stand out from the selected articles, considering the type of research done and sample sizes. Firstly, there are no multicenter studies among the articles included. Single-center study data might be biased by case complexity and imaging vendor. On the contrary, multicenter studies allow for more representative data as multiple outcomes, different vendors, and catheterization settings are investigated [33]. Secondly, the articles have a retrospective nature that the authors properly stated to be a limitation [3, 4, 6, 14, 27]. If a prospective design is feasible, a retrospective design should not be used. A prospective study could investigate the value and radiation dose of 3DRA versus CA in the same patient sample, whereas a retrospective study must examine which cases resemble each other enough to increase the precision of the comparison [34]. Thirdly, ten articles are merely of descriptive nature as they describe the procedure using 3DRA in a single case or case series of maximum eight patients, but do not compare or analyze the data.

It is also striking that the documentation of the results is not universal. To begin with, not all articles documented or discussed radiation, whereas others qualitatively researched 3DRA and compared it to CA by radiation dosages, contrast, and fluoroscopy time. Before comparisons can be made, a standardized way of reporting these data is necessary. Furthermore, radiation dosages are reported in different ways: DAP, ED, or AK. Concerning radiation and the possible negative effects on children, the ED is the best representative of the actual radiation the patient is subjected to because it is a weighted average of the doses to radiosensitive organs in the body [35]. Though DAP, the product of radiation dose and exposed patient surface [3] is more often reported. The ED can be calculated by using the DAP and then applying the Monte Carlo program [33]. DAP itself has also shown to correlate with ED and is therefore relatively reliable as a measurement [27]. Although ED might be the best representative, it is advised to both record the DAP and calculate the ED.

Another important point is that the sample sizes, age ranges, vendors, and types of interventions differed per study, which influenced the compatibility of the articles that are included in this review. If the methods and intentions of these studies had been more congruous, the results might have been a better representation for the use of 3DRA. The study by Haddad and colleagues expresses that children require different radiation protocols because they vary in size and even adults with different proportions receive different amounts of radiation [19]. Therefore, it is illogical that an adult would be included in the same research sample as a one-year-old child. Especially, considering that the DAP values are subsequently calculated into a mean that is supposedly a representative of a population with a mean age < 10 years [6, 12, 14, 22]. Moreover, many of the other articles had a patient population varying between 0 and 19 years of age. While this is technically a pediatric population, the problems with patients’ size and weight remain. A few studies demonstrate that a substantial sample size with a homogeneous diagnosis and age is possible and attains significant results [3, 17]. It is thus strongly urged that the homogeneity of the age group is taken seriously in pediatric research, particularly those concerning contrast and radiation exposure. In that case, the results of these studies could be used to find correlations and even make conclusions about the use of 3DRA in the pediatric population.

All the articles mentioned the high quality of 3DRA; it seems to become progressively popular and many articles speculate about 3DRA becoming the standard imaging technique for many procedures [4, 8, 28]. While the imaging might be of superior quality, the studies barely document complications, adverse events, or quality of life due to the use of 3DRA. Starmans and colleagues clearly report the complications observed in their population. Only one of the 16 complications in the 3DRA group, being transient right bundle branch block after right ventricular pacing, could be related to 3DRA [3]. Pockett et al. discussed the fact that none of their patients suffered from a major or catastrophic conduit disruption, whereas the reported incidence is 1.4–2.7% with conventional angiography. They related this to stent stabilization of conduit walls and increased structural integrity of the conduit using the 3DRA technique, which limited the risk of conduit tears and ruptures from initial balloon dilation [28]. Granted that their sample size only consisted of 31 patients, these results are not definitive. However, it is important for all articles to consider the implications, positive or negative, that 3DRA can have on the patients, whether or not the imaging is of superior quality and if the contrast and radiation exposure can be reduced.

### Future Perspective

Future research should investigate the true advantages of 3DRA and how exactly it benefits the patients. Therefore, large, prospective, homogenous, multicenter studies are necessary on children with one type of congenital heart disease. In addition, these studies should universally document the outcomes, including radiation dosages, procedural and fluoroscopy time, contrast dye consumption, adverse events, final clinical results, and quality of life in patients treated with the use of 3DRA to make a proper comparison possible. Eventually, the results obtained from these studies could be translated to generalized protocols that would tackle the learning curves of inexperienced institutions.

### Limitations

A limitation of this review is that sole inclusion of articles discussing 3DRA in children with congenital heart disease was not possible. In addition, comparison of the results of 3DRA with CA in context of a meta-analysis was neither fair nor possible, as the studies were too heterogeneous and the patients had different age ranges and diagnoses.

## Conclusion

Even though 3DRA is already a greatly valued diagnostic and therapeutic technique, the literature misses homogeneous, multicenter, prospective research that records its data in a standardized manner to ensure proper analysis of this research. Currently, research focuses on the novelty of 3DRA as a tool in pediatric patients with CHD and state that 3DRA should be preferred over CA because of the benefits to the patients. However, future research should investigate the true advantages of 3DRA and how exactly it benefits the patients.
